# Improvement of Functional Recovery of Donor Heart Following Cold Static Storage with Doxycycline Cardioplegia

**DOI:** 10.1007/s12012-013-9231-1

**Published:** 2013-10-09

**Authors:** Evren Ozcinar, Esma N. Okatan, Erkan Tuncay, Sadik Eryilmaz, Belma Turan

**Affiliations:** 1 Department of Cardiovascular Surgery, Ankara Diskapi Training and Research Hospital, Ministry of Health, 06330 Ankara, Turkey; 2 Department of Biophysics, Faculty of Medicine, Ankara University, Ankara, Turkey; 3 Faculty of Medicine, Cardiovascular Surgery Heart Center, Ankara University, 06100 Ankara, Turkey

**Keywords:** Cardioplegia, Cold storage, Heart function, Ischemia, Reperfusion, Matrix metalloproteinases, Apoptosis

## Abstract

Injury to the donor heart during cold preservation has a negative impact on graft survival before transplantation. This study aims to examine whether doxycycline, known as an MMP-2 inhibitor, has a positive effect on donor heart preservation via its antioxidant action when added to standard preservation solution. Hearts were obtained from 3-month-old male Wistar rats and randomly divided into three groups: hearts stored for 1 h at 4 °C (1) with doxycycline preservation solution (DOX cardioplegia) with low Ca^2+^; (2) with standard cardioplegia with low Ca^2+^; and (3) unstored hearts. All hearts were perfused in working mode, arrested at 37 °C, removed from the perfusion system, reattached in Langendorff perfusion system, and converted to working mode for 1 h. At the end of the storage period, hearts preserved in DOX cardioplegia had significantly less weight gain than those preserved in the standard cardioplegia. DOX cardioplegia-induced preservation resulted in significantly higher heart rates and better recovery quality during reperfusion in aortic flow compared to the standard cardioplegia group. Recovery in the left ventricular function and Lambeth Convention Arrhythmia scores during 1 h reperfusion were also significantly better in the DOX cardioplegia group. Biochemical data showed that DOX cardioplegia prevented an increase in MMP-2 activity and blocked apoptosis through increased activity of the pro-survival kinase Akt in the donor heart homogenates. DOX cardioplegia also led to a balanced oxidant/antioxidant level in the heart homogenates. This is the first study to report that cardioplegia solution containing doxycycline provides better cardioprotection via the preservation of heart function, through its role in controlling cellular redox status during static cold storage.

## Introduction

The cardiac transplantation is a life-saving procedure for patients with severe heart failures. However, its clinical application remains limited due to lack of donor heart availability [1], and the method currently used for donor heart preservation, the static cold storage (+4 °C), allows a very short preservation time of only 4–6 h outside the body [2]. These limitations have triggered a search for improved methods of preservation that could allow for prolonged storage of donor hearts. Although continuous machine perfusion of donor hearts has been proposed as an alternative to cold static storage, multicentered clinical investigations showed continuous machine perfusion to be an expensive technique, and due to its small market size, there is little commercial interest in developing its devices.

Continuous perfusion of harvested hearts with oxygen and metabolic substrates was reported to help maintain myocardial integrity during organ transport, therefore providing better support in preservation [3–5]. While there has been ample research into improving the quality of donor hearts and prolonging the preservation time, most previous studies had conflicting results due to the use of small animals with significantly different anatomies and physiologies from those of humans. Current preservation protocols use hypothermic arrest and simple storage, using a variety of crystalloid-based cardioplegic and preservation solutions [6, 7]. These techniques limit organ procurement and safe storage time to 4–6 h. A recent study showed marked improvements in donor heart function after 8 h of cold static storage, using normokalemic, adenosine, lidocaine, melatonin, and insulin preservation solution for the isolated rat heart [8].

Cold storage is a simple, inexpensive, and reliable technique for preserving donor hearts during the ex vivo transport period [9]. However, several obstacles limit better preservation of donor hearts during the preservation interval [10]. Longer arrest times easily lead to donor heart damage and early graft dysfunction [11]. Furthermore, there is a well-established risk for primary graft dysfunction when using hearts from extended criteria donors. Depending on the duration of the ischemic period, ATP consumption, ion-homeostasis, and free radical-mediated reperfusion injury also affect the postoperative myocardial dysfunction [12]. Therefore, strategies for improved preservation are necessary, particularly for more effective long-term preservation of organs.

During the last decade, research focused on a group of enzymes known as matrix metalloproteinases (MMPs), which are important mediators in cardiovascular pathologies associated with enhanced oxidative stress. The MMPs are synthesized in a latent form and are activated by proteolytic or conformational changes similar to those induced by oxidative stress [13]. MMPs have also been shown to play significant intracellular roles, including the degradation of extracellular matrix components and long-term tissue remodeling [14]. In isolated perfused heart studies, MMP inhibition was shown to reduce ischemia/reperfusion (I/R)-induced troponin I-degradation and significantly improve the recovery of mechanical function [15].

The tetracycline class antibiotics have a distinct additional pharmacological property, independent of their antibacterial action, in relation to MMPs: Doxycycline (DOX), a member of the tetracycline family antibiotics, has been shown to inhibit both expression and activity of MMP-2 [13] and to preserve cardiac function against I/R injury in the heart [15]. In addition, recent reports further indicate that DOX directly inhibits the cysteine protease activity, and indirectly inhibits the serine protease activity through the inhibition of MMP-mediated degradation of endogenous serine protease inhibitors [13].

We have previously demonstrated that in vivo DOX treatment of diabetic rats preserved both cardiac and aortic functions due to its antioxidant-like action [16]. Therefore, in the present study, we hypothesized that cold static storage of the donor heart with DOX cardioplegia may prevent I/R-induced injuries, and thus preserve cardiac function, by prolonging the preservation period. We used an isolated perfused heart model, in which hearts were perfused in the working state and preserved in the modified Krebs–Henseleit solution for 1 h with either DOX preservation solution or standard preservation solution at +4 °C. This is the first study to report that cardioplegia solution containing DOX provides better cardioprotection via the preservation of heart function through its role in controlling cellular redox status as well as by blocking apoptosis through increased activity of the pro-survival kinase Akt in the donor heart homogenates during static cold storage.

## Materials and Methods

### Experimental Animals

All animals were handled in accordance with the *Guide for the Care and Use of Laboratory Animals* (National Institutes of Health, Bethesda, MD). The protocol of the study was approved by the Local Ethics Committee on Animal Experiments of the Ankara University (Approval no. 2009-44-198).

Hearts of 3-month-old Wistar male rats weighing 250–300 g were used. Rats were housed under a 12-h/12-h light/dark cycle with food and water provided ad libitum during the experimental protocol.

### Perfusion Medium

Hearts were rapidly excised from pentobarbital-anesthetized (30 mg/kg body weight, intraperitoneal) rats and briefly submerged in ice-cold Krebs–Henseleit buffer. The composition of the buffer was as follows: 118 mmol/L NaCl; 4.7 mmol/L KCl; 1.2 mmol/L KH_2_PO_4_; 1.2 mmol/L MgSO_4_; 1.8 mmol/L CaCl_2_; 25 mmol/L NaHCO_3_; 10 mmol/L glucose; 0.5 mmol/L EDTA; 9 mmol/L mannitol; and 1 mmol/L Dulbecco’s modified Eagle’s medium (DMEM). The final pH was adjusted to 7.4, and the resultant solution was gassed continuously with a mixture of 95 % O_2_ and 5 % CO_2_.

### Isolated Heart Storage

Isolated hearts were stored with an added low CaCl_2_ (0.5 mmol/L) and/or added DOX (100 μmol/L), MMP inhibitor doxycycline, and gassed with a mixture of 95 % O_2_ and 5 % CO_2_ in modified Krebs–Henseleit buffer. Prior to the perfusion protocol, isolated hearts were preserved in the modified Krebs–Henseleit solution with iced packages for 1 h, with either DOX preservation solution or standard preservation solution at +4 °C.

### Langendorff Perfusion of Isolated Hearts

Spontaneously beating hearts were perfused via their aortas at a constant pressure of 60 mmHg with Krebs–Henseleit buffer at 37 °C after the storage periods. A water-filled latex balloon connected to a pressure transducer was inserted into the left ventricle through an incision in the left atrium and through the mitral valve, and the volume was adjusted to achieve a stable end-diastolic pressure (8–12 mmHg). Heart rate (HR), arrhythmias (analyzed according to Lambeth Convention Arrhythmia scores), and left ventricle developed pressure (LVDP) were monitored on a polygraph. Coronary flow was measured with an in-line ultrasonic flow probe (Transonic Systems, Inc.) positioned proximal to the perfusion cannula. Weight gains of the hearts during preservation were assessed before and after the storage period. The hearts were maintained to a steady state of coronary flow. All hearts were stored at −80 °C until protein analysis following the electrophysiological procedure was performed.

### Preparation of Heart Homogenates

Frozen hearts were crushed at liquid nitrogen temperature and then homogenized in 50 mmol/L Tris–HCl (pH 7.4) containing 3.1 mmol/L sucrose, 1 mmol/L DTT, 10 μg/mL leupeptin, 10 μg/mL soybean trypsin inhibitor, 2 μg/mL aprotinin, and 0.1 % Triton X-100. The homogenates were centrifuged at 10,000×*g* at 4 °C for 10 min. The supernatants were collected as cytosolic fractions, stored at −80 °C, and then were used to measure MMP-2 (tissue inhibitor of matrix metalloprotein), phospho-Akt, Akt, Bcl-2 (an apoptosis inhibitor), and Bax (an apoptosis promoter) protein levels. Protein contents in homogenates were analyzed by using Bradford Protein Assay (Bio-Rad), and bovine serum albumin was used as a protein standard.

### Gelatin Zymography

Gelatin zymography to measure MMP activity was performed as described previously [16]. Non-reduced proteins were loaded onto an 8 % polyacrylamide gel containing gelatin. Gelatinolytic activities were detected as transparent bands against the background of Coomassie blue-stained gelatin. Tissue homogenates (20 μg) were loaded onto gels to visualize MMP-2 activity. To quantify MMP-2 activity, zymograms were imaged by a Raytest camera attached to a computer with AIDA software (Germany). Gelatinolytic activity was identified using cell culture medium of LTK8 fibroblast cell line as a positive control. Zymograms were digitally scanned, and intensities of the bands were quantified using SigmaGel (Jandel) and reported as a normalized form with respect to their controls.

### Western Blotting

Protein expression levels of MMP-2, phospho-Akt, Akt, Bcl-2, and Bax were determined by Western blot analysis. Equal amount of proteins from tissue homogenates were loaded and separated on 8 % sodium dodecyl sulfate polyacrylamide gel electrophoresis (SDS-PAGE) gels under reducing conditions. After electrophoresis (150 V, for 3 h, at 20 °C), samples were electroblotted onto a PVDF membrane by wet transfer in Towbin buffer (25 V, for 2 h). The β-actin levels in the gel were identified as a loading control for MMP-2-specific antibodies. Immunoreactive protein bands were visualized by using an ECL plus detection system.

#### Measurement of Total Oxidant and Total Antioxidant Status in Heart Homogenates

Total oxidant and total antioxidant status in the heart homogenates were measured by using commercial kits (Rel Assay Diagnostics). Total oxidant status measurement is based on the oxidation of the ferrous ion–*o*-dianisidine complex to ferric ion by the oxidants present in the samples. The ferric ion makes a colored complex with xylenol orange in an acidic medium. The color intensity, measured spectrophotometrically, is related to the total amount of oxidant molecules present in the samples. The assay is calibrated with hydrogen peroxide (H_2_O_2_), and the results are expressed in terms of μmol H_2_O_2_ equivalent per liter. Principle of total antioxidant status assay is based on the oxidation of the reduced 2,2′-azino-bis (3-ethylbenz-thiazo-line-6-sulfonic acid) (ABTS) molecule, which oxidized to ABTS^+^ using H_2_O_2_ alone in acidic medium (the acetate buffer 30 mmol/L, pH 3.6). In the acetate buffer solution, the concentrate (deep green) ABTS^+^ molecules stay more stable for long time. While it is diluted with a more concentrated acetate buffer solution at higher pH values (the acetate buffer; 0.4 mol/L, pH 5.8), the color is spontaneously developed and slowly bleached. Antioxidants present in the sample accelerate the bleaching rate to a degree proportional to their concentrations. This reaction can be monitored spectrophotometrically, and the bleaching rate is inversely related with total antioxidant status (TAS) of the samples. The reaction rate is calibrated with Trolox standard (an analog of vitamin E) and is expressed as mmol Trolox equivalent per liter.

### Statistical Analysis

Data were expressed as mean ± SEM. Statistical analysis (GraphPad Prism) was performed using Wilcoxon matched-pairs signed rank test, one-way ANOVA, or Mann–Whitney *U* test as appropriate. A *p* value of <0.05 was considered statistically significant.

## Results

### DOX Preservation Solution Preserves Heart Function During Cold Static Storage

**Fig. 1:**
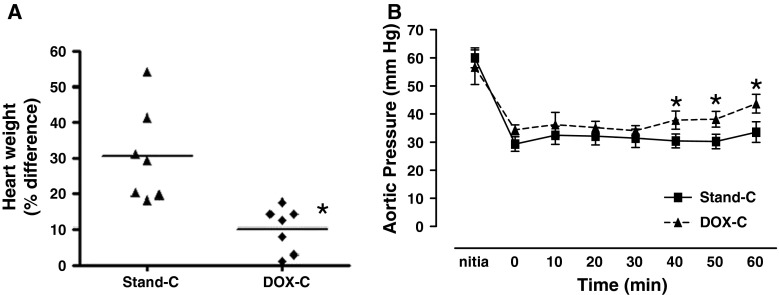
Using DOX preservation solution (DOX cardioplegia) preserves donor heart weight and aortic pressure during the 1 h of reperfusion after 1-h cold static storage. **a** Heart weights are expressed as percentage differences between the values of before and after cold (+4 °C) static storage (for 1 h) with either DOX cardioplegia (DOX-C) or standard cardioplegia (Stand-C). **b** Time-dependent recovery pattern of aortic pressure (coronary perfusion pressure) during 1 h of reperfusion after cold static storage (with either DOX cardioplegia or standard cardioplegia) with respect to its initial value. Values are mean ± SEM for *n* = 7–9 rats/protocol. *Asterisks* indicate significant differences between the values stored with DOX cardioplegia and standard cardioplegia (*p* < 0.05) by one-way ANOVA (in **a**) and Mann–Whitney *U* test (in **b**)

In order to investigate the effect of cold static storage method on myocardial edema, we recorded and compared the weights of hearts preserved in the DOX preservation solution (DOX cardioplegia group) with those of the standard preservation solution. As shown in Fig. 1a, the average heart weights following 1-h cold static storage were significantly less in the DOX cardioplegia group.

Recovery of aortic flow was monitored by measuring the aortic pressure of Langendorff-perfused hearts during the 1-h reperfusion period, following 1-h cold static storage with standard or DOX preservation solution. Figure 1b shows the time course of aortic pressure recovery during 1-h reperfusion period. The cold static storage with standard preservation solution induced about 50 % decrease in the aortic pressure measured and kept stable those depressed pressure during 1-h reperfusion period, whereas the aortic flow of the DOX cardioplegia group significantly recovered after the first 30 min of the reperfusion period.

**Fig. 2:**
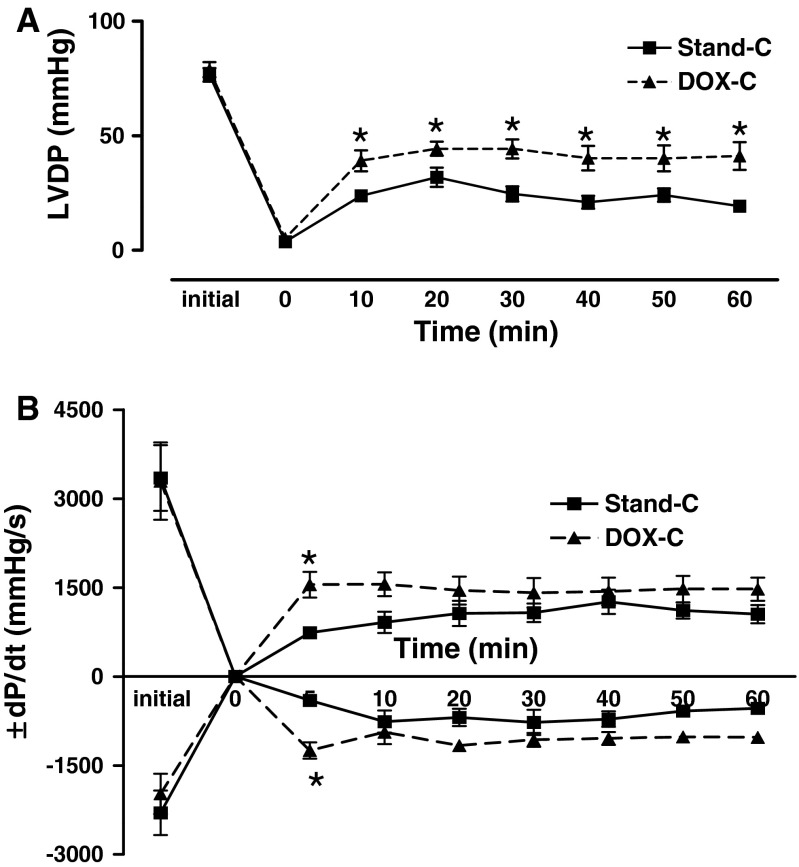
DOX cardioplegia presents cardioprotection in the left ventricular pressure changes in donor heart during the 1-h reperfusion after 1-h cold static storage. Time-dependent recovery patterns of left ventricular developed pressure, LVDP **a** and the rates of changes in the developed pressure (±d*P*/d*t*) **b** after cold (+4 °C) static storage (for 1 h) comparison with the values before the storage with either DOX cardioplegia (DOX-C) or standard cardioplegia (Stand-C). Values are mean ± SEM for *n* = 7–8 rats/protocol. *Asterisk* indicates significant difference between the values stored with DOX cardioplegia and standard cardioplegia (*p* < 0.05) by one-way ANOVA

Figure 2 shows the left ventricular developed pressure (LVDP; 2a) and the rates of pressure changes (±d*P*/d*t*_max_; 2b) at baseline (the value before cold static storage) and during reperfusion of donor hearts. The recoveries both in LVDP’s and their derivatives (±d*P*/d*t*_max_) were found to be significantly improved in the DOX cardioplegia group, compared to the standard preservation solution group.

### Effects of DOX Preservation Solution on Recovery of Heart Rates and Lambeth Convention Arrythmia Scores

**Fig. 3:**
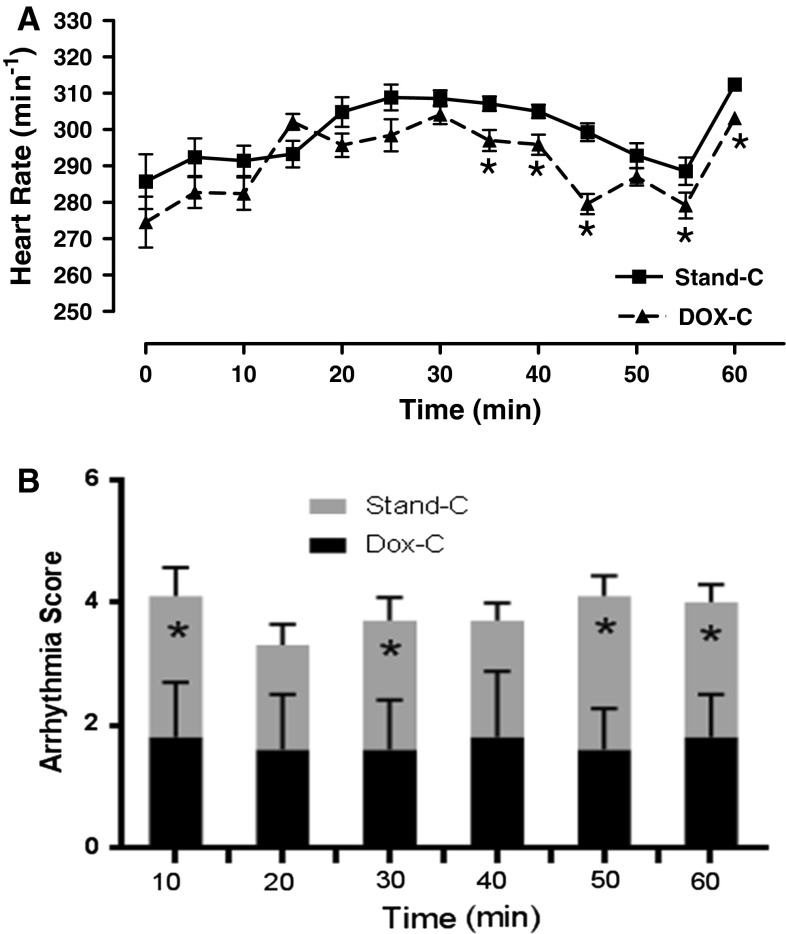
Effect of using DOX cardioplegia on heart rate and Lambeth Convention Arrhythmia (LCA) scores during the 1-h reperfusion after 1-h cold static storage. Recovery patterns (in a time-dependent manner) of heart rate (**a**) and LCA scores (**b**) during 1-h reperfusion measured every 10 min after 1-h cold static storage of donor hearts. Comparison with the values before the storage with either DOX cardioplegia (DOX-C) or standard cardioplegia (Stand-C). The arrythmia scores of the hearts were recorded with bipolar ECG by using two electrodes. Values are mean ± SEM for *n* = 7–9 rats/protocol. *Asterisk* indicates significant difference between the values stored with DOX cardioplegia and standard cardioplegia (*p* < 0.05) by one-way ANOVA (in **a**) and Mann–Whitney *U* test (in **b**)

The time course of spontaneous heart rates of donor hearts during the 1-h reperfusion period following 1-h cold static storage with or without DOX preservation solution is given in Fig. 3a. The baseline values (or control values without any storage) of the heart rates of donor hearts ranged from 320 to 360 beats/min^−1^. As shown in Fig. 3a, the recovery in the heart rate during the 1-h reperfusion period was better in the DOX cardioplegia group than the standard preservation solution group, even though the time course of these two groups overlapped during the first 30 min of the reperfusion period.

Figure 3b shows the Lambeth Convention Arrythmia (LCA) scores during the 1-h reperfusion period (presented as the value in every 10 min). The LCA scores of donor hearts were recorded using bipolar ECG with two electrodes. The hearts spontaneously beat during reperfusion to mimic donor hearts. In the 1-h reperfusion period, the LCA scores were monitored and presented as mean (±SEM). Compared to those of the unstored group (data not given), the LCA scores of these two groups significantly increased during the reperfusion period. The LCA scores of the DOX cardioplegia group were significantly better, compared to standard preservation solution group.

### DOX Preservation Solution Controls MMP-2 Activity in Donor Heart Homogenates During Cold Static Storage

**Fig. 4:**
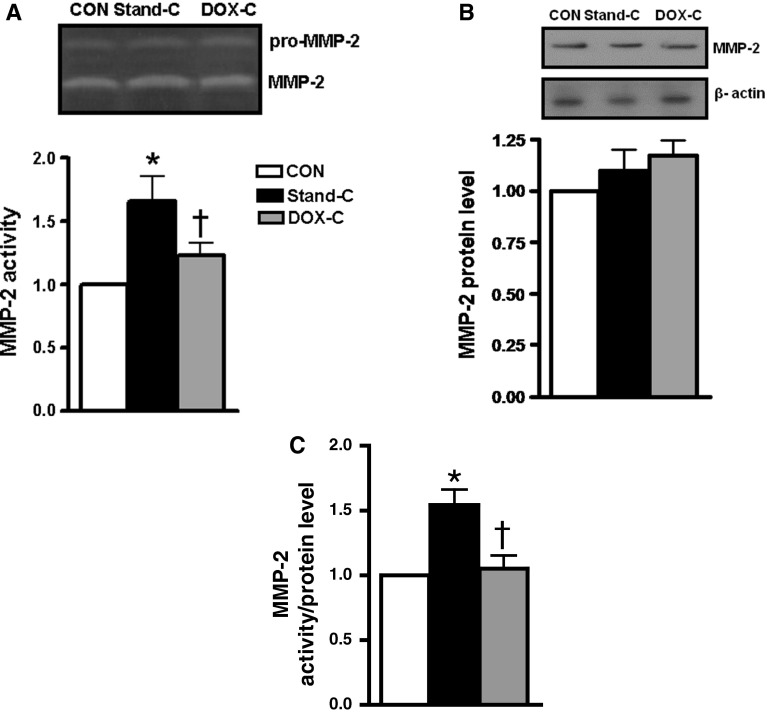
DOX cardioplegia during 1-h cold static storage of donor heart prevents increase in MMP-2 activity measured in the myocardium. **a** *Top* for representative zymogram of 72-kDa MMP-2 activity and at *bottom* for quantification of 72-kDa MMP-2 activity. **b** *Top* for representative Western blotting of 72-kDa MMP-2 protein level and at *bottom* for quantification of 72-kDa MMP-2 protein level with respect to 43-kDa β-actin. **c** The ratio of 72 kDa MMP-2 activity to 72 kDa MMP-2 protein level. *Bars* represent mean ± SEM, *n* = 5–6 homogenates/group/protocol for unstored hearts (CON), stored hearts with DOX cardioplegia (DOX-C) or standard cardioplegia (Stand-C) for 1 h at +4 °C. *p < 0.05 versus CON group, ^†^ *p* < 0.05 versus Stand-C group by one-way ANOVA

Analysis of gelatin zymography performed in the homogenized heart tissues of the DOX cardioplegia group revealed marked gelatinolytic MMP-2 activity at 72 kDa (Fig. 4a, upper part) compared to those of the unstored hearts. Although Western blot analysis of the heart homogenates revealed a slight trend toward increased MMP-2 protein content after 1-h cold static storage either with or without DOX preservation solution, they were not statistically significant from that of the unstored group (Fig. 4b). As shown in Fig. 4c, while the ratio of MMP-2 activity to its protein level is significantly higher in the standard preservation solution group compared to that of the unstored group, it was found to be normalized by cold static storage using DOX preservation solution.

### Doxycycline Reverts Storage-Induced Impairment of Survival Pathways During Cold Static Storage of Donor Heart

**Fig. 5:**
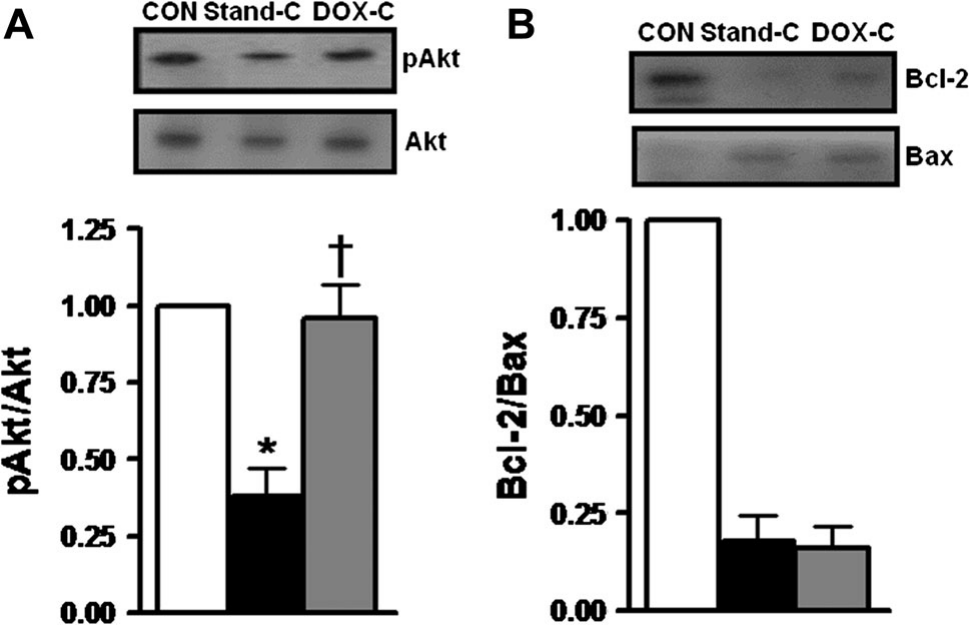
Doxycycline preservation solution reverts impairment of survival pathway during cold static storage of donor hearts. Phosphorylated Akt (pAkt) to total Akt ratio (**a**) and Bcl-2/Bax ratio (**b**) determined by Western blot in cardiac tissue homogenates. *Bars* represent mean ± SEM, *n* = 5–6 homogenates/group/protocol for unstored hearts (CON), stored hearts with DOX cardioplegia (DOX-C) or standard cardioplegia (Stand-C) for 1 h at +4 °C. *p < 0.05 versus CON group, ^†^ *p* < 0.05 versus Stand-C group by one-way ANOVA

In order to test a possible positive contribution of the DOX preservation solution into apoptosis signaling pathway during the 1-h cold static storage of donor heart, we first measured the phosphorylation level of Akt (pAkt) with respect to its protein level in the homogenates. As shown in Fig. 5a, while the ratio of pAkt to Akt in donor heart homogenates kept in the standard preservation solution was markedly lower compared to that of the unstored ones, it was fully preserved in the DOX cardioplegia group.

We also examined another factor as a marker of survival pathway, Bcl-2/Bax ratio, a marker of apoptosis. As shown in Fig. 5b, DOX preservation solution did not have a significant effect on the increased level of Bcl-2/Bax ratio measured in the donor heart following 1-h cold static storage.

### DOX Preservation Solution During Cold Static Storage of Donor Hearts Preserves Myocardial Total Antioxidant Capacity

**Fig. 6:**
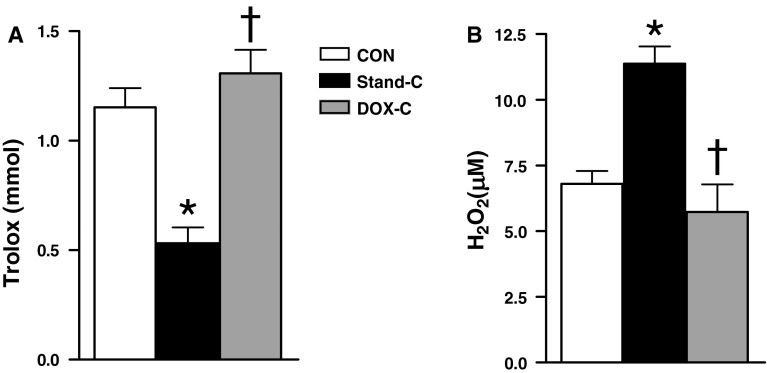
DOX preservation solution during cold static storage of donor hearts preserves total antioxidant capacity of the myocardium. Total antioxidant status measured with respect to Trolox (**a**) and total oxidant status measured with respect to H_2_O_2_ (**b**) in heart homogenates of unstored hearts (CON), stored hearts with DOX cardioplegia (DOX-C) or standard cardioplegia (Stand-C) for 1 h at +4 °C. *Bars* represent mean ± SEM, *n* = 5–6 homogenates/group/protocol. *p < 0.05 versus CON group, ^†^ *p* < 0.05 versus Stand-C group by one-way ANOVA

A well-established method to demonstrate oxidative stress markers in any tissue is to measure both total oxidant status (TOS) and total antioxidant status (TAS) [16]. To explore the balance between TOS and TAS in donor hearts after 1-h cold static storage, we measured their levels in the heart homogenates of all three groups. While TOS level was higher, TAS level was lower in the donor hearts after 1-h cold static storage with standard preservation solution compared to those of both unstored and the DOX cardioplegia groups (Fig. 6a, b, respectively). Therefore, this suggests that using DOX preservation solution during cold static storage of donor hearts can preserve the balance between TOS and TAS in donor myocardium.

## Discussion

The present study demonstrated that donor heart preservation solution containing doxycycline (DOX cardioplegia) provides much better cardioprotection than standard cardioplegia during the 1-h reperfusion following 1 h of cold static storage. Better cardioprotection was evidenced by normalized left ventricular heart function and Lambeth Convention Arrhythmia scores, which were further supported by normalized MMP-2 activity, and partly with blockage of apoptosis through increased activity of pro-survival kinase Akt in the donor heart homogenates.

Composition of the perfusion solution is one of the key factors for the success of the cold static preservation, which is still the most widely used technique to preserve donor hearts [17]. However, despite extensive research, the optimal preservation solution is yet to be defined. In fact, Demmy et al. [18] determined use of 167 different types of heart preservation solutions for perfusion in the USA alone. The use of suboptimal solutions, imperfect for minimizing certain important functional alterations in the donor heart, may lead to cardiac allograft dysfunction [19].

Oxidative stress-associated alterations in several intracellular pathways have been implicated in the pathophysiology of severe donor heart damage during reperfusion. As shown in earlier studies, a fundamental pathway for cardiac damage during reperfusion includes marked increases in the amount of superoxide anion, hydrogen peroxide, and possibly singlet oxygen production [20]. In line with these findings, it has been demonstrated that a Bretschneider’s solution, developed as a cardioplegic solution in routine cardiac surgery, could effectively reduce energy requirements and prevent damages during reperfusion, due its role as a strong reducing agent of hydroxyl radicals and reactive oxygen species (ROS), leading to improved myocardial protection via controlling cellular oxidative stress levels [21, 22]. Therefore, preventing or at least to controlling increases in the oxidative stress levels of donor hearts during cold storage seems to be a crucial part of heart transplantation in cardiac surgery. This present study reports an effective improved myocardial protection during reperfusion whereby donor hearts are stored in a cardioplegia containing DOX (DOX cardioplegia) for 1 h at +4 °C.

Previous research has indicated the need for greater understanding of the role of Akt’s mechanism, one of the essential mechanisms for surgical and other clinical sciences, in the protection of heart preparations during ischemia–reperfusion [23]. Our present data demonstrated that DOX preservation solution played an important role in the apoptosis signaling pathway by increasing the phosphorylation level of pro-survival kinase Akt during 1-h cold storage of donor hearts. However, at the same time, dramatically increased Bcl-2/Bax ratio in the same homogenates could not be preserved. The Akt kinase regulates processes of cellular proliferation and survival, including inhibition of transcriptional functions of Forkhead box-O transcription factors (FoxOs) and contribution to cell survival, growth, and proliferation via FoxOs phosphorylation by Akt. It should be noted that heart failure continues to be one of the most important causes of morbidity and mortality due to increased cell death and limited capacity of myocyte renewing. Akt is considered the central regulator of cardiomyocyte survival after severe in vivo and in vitro ischemic lesions [24]. Akt activation (phosphorylation) has suppressed apoptosis induced by hypoxia in a variety of cellular models including ventricular myocytes [25] and reduced apoptosis and the size of the infarct area in hearts [26]. Bax, a pro-apoptotic protein, and Bcl-2, anti-apoptotic, participate in the intrinsic pathway of apoptosis with opposite roles. While Bax activation results from an increase in mitochondrial permeability, Bcl-2 levels are increased by growth factors and other survival signals. Akt has also been shown to have a critical role in activating a transcription factor of cAMP response element-binding protein, a positive regulator of Bcl-2 expression [27]. The ratio between pro- and anti-apoptotic factors is widely considered to be the main trigger initiating the apoptotic pathway. Our data show dramatic increase in Bcl-2/Bax ratio in the donor heart with DOX preservation solution during 1-h cold storage, and this does not fully support the above idea. However, cardioprotection with this preservation solution was made possible at least in part through an increase in Akt activation (phosphorylation). Further studies are needed to clarify precisely how a DOX cardioplegia may protect a donor heart during cold static storage.

The increase in production of ROS and generation of oxidative stress, which may result from impairment of several intracellular signal transduction cascades, can cause modulation of MMPs in several cell types [28]. Concurrently, a decrease in endothelial NO availability is reported to induce a significant increase in the activity of MMPs [29]. Doxycycline, a member of the tetracycline family of antibiotics, does not only have antimicrobial mechanisms but also inhibits connective tissue breakdown [30]. Doxycycline with low dose usages has been demonstrated to exert anti-inflammatory and antioxidant activity [31, 32]. Accordingly, it has also been shown that doxycycline inhibited NO production and protected some tissues against doxorubicin-induced oxidative stress as well as apoptosis in mouse model [33, 34]. Moreover, minocycline, a semisynthetic derivative of tetracycline, showed a marked protective effect against oxidative stress-induced injury due to its antioxidant properties as a free radical scavenger [31, 32]. Studies with doxycycline further demonstrated attenuation of protein aggregation in cardiomyocytes, improvement in the survival of a mouse model of cardiac proteinopathy, and inhibition of MMPs to be effective therapeutic interventions in the management of acute pulmonary thromboembolism [35, 36]. Our present data with doxycycline are in line with these previously published data on preventive action of doxycycline performed in different pathological heart models.

Matrix metalloproteinases are a family of proteases best known for their capacity to proteolyse several proteins associated with extracellular matrix. Their increased activity contributes to the pathogenesis of several cardiovascular diseases including ischemia/reperfusion injury in the heart [15, 37]. MMP-2, in particular, is now considered to be also an important intracellular protease, having ability to proteolyse specific intracellular proteins in cardiac muscle cells and thus reduce contractile function [36]. Doxycycline has been frequently used as an important MMP inhibitor independent of its antimicrobial property. In the present study, when we added doxycycline into heart preservation solution during cold static storage of donor heart, we observed a significantly better recovery process in the donor heart function during the reperfusion period compared to that of the standard perfusion solution. Furthermore, we obtained a balanced oxidant/antioxidant ratio and normalized MMP-2 activity in the heart homogenates stored with DOX cardioplegia. Therefore, our biochemical data indicate that this cardioprotection with doxycycline may have emerged due to not only its MMP-2 inhibitor action but also its strong antioxidant action [16].

A common problem with the cold storage preservation has been myocardial edema formation during reperfusion, which drives graft dysfunction and leads to failure. Buttler et al. [38] investigated the relationship between edema and cardiac dysfunction by inducing ischemia versus edema alone in isolated cardiomyocytes and Langendorff-perfused hearts. Edema-induced dysfunction was mild in both cellular preparation and at the whole organ level, which suggested a need for reappraisal of the edema-mediated dysfunction after cardiac surgery in the patients. In our study, a significant reduction in the myocardial edema was observed in hearts preserved with DOX cardioplegia during cold static storage. In line with our findings, Fert-Bober et al. [39] also showed that MMP inhibitors prevented edema formation by reducing damage to the endothelial barrier function of the cells.

Ventricular fibrillation is a serious ischemia–reperfusion-induced complication. Although short action potential period of rat heart seems to pose a disadvantage, ischemia-induced ventricular fibrillation rate is generally high [40, 41]. The arrhythmia and ventricular fibrillation incidences usually increase after heart transplantation. In our study, mimicking heart transplantation preservation model, atrioventricular nodes of the hearts were left intact and they continued beating spontaneously during perfusion. During 60-min reperfusion period, the arrhythmia incidence and its period were significantly better in the DOX cardioplegia group, compared to the standard preservation solution group. This is the first data available in the literature about the relationship between MMP activation and arrythmia incidence in different heart preparations. These data suggest the need for further research into doxycycline’s potential use as an antiarrhythmic drug in cardiac dysfunction in general. Indeed, using new chemical agents to improve cardioprotection in donor heart transplantation is a well-recognized strategy for cardiac surgery. An ideal preservation solution should provide prolonged, safe, and predictable preservation of donor organs. Supporting this hypothesis, Yang and Yu [42] obtained prolonged donor heart preservation with pinacidil, due to its cardioprotection with better energy preservation and improvement in the myocardial recovery after deep hypothermia and prolonged ischemic storage.

### Limitations

The present study involves an experimental design performed under in vitro condition at the organ and its tissue level. Further research will be necessary to evaluate the effect on whole cardiac function under in vivo condition in terms of the dosage and time period of doxycycline use.

## Conclusion

In conclusion, our study suggests that doxycycline is a good candidate for the heart preservation solution during cold static storage of donor hearts before transplantation. Doxycycline seems to contribute to the reduction in oxidants, thereby controlling oxidative stress levels in the stored hearts. Our findings point to its therapeutic potential, partly due to this antioxidant action, for protecting myocardium against oxidative stress-induced damage. We believe doxycycline may play a strategic role in improving the cardioprotection during reperfusion following ischemia, thereby contributing to prevention of heart injury, which presents a high risk of mortality and morbidity in the transplanted subjects [43, 44]. The underlying mechanism of cardioprotection with doxycycline in donor heart preservation with cold static storage requires further investigations.
